# To explore the risk factors of lymphovascular invasion in patients with upper tract urothelial carcinoma and construct a prediction model

**DOI:** 10.3389/fonc.2025.1568774

**Published:** 2025-03-25

**Authors:** Qinghui Li, Pengtao Wei, Yanjie Kang, Xiaohui Li, Han Zhang, Jinhui Yang, Jiantao Sun

**Affiliations:** Department of Urology, Luoyang Central Hospital Affiliated to Zhengzhou University, Luoyang, China

**Keywords:** upper tract urothelial carcinoma, nephroureterectomy, lymphovascular invasion, risk factors, prediction model

## Abstract

**Background and objective:**

To explore the risk factors and construct a prediction model of the lymphovascular invasion (LVI) in patients with upper tract urothelial carcinoma (UTUC).

**Methods:**

Clinical data of 143 UTUC patients treated in our hospital during Jan. 2010 and Dec. 2022 were retrospectively analyzed. The patients were divided into LVI positive group and LVI negative group according to the postoperative lymphovascular conditions. Kaplan-Meier method was used to evaluate the overall survival (OS) and cancer-specific survival (CSS) of the two groups, and the survival curve was drawn. The correlation between LVI and inclusion indexes was analyzed using univariate and ultivariate. A prediction model was established and receiver operating characteristic (ROC) curve was drawn to analyze the diagnostic value.

**Results:**

The median survival time of LVI positive patients was 78 months (95%CI 44.47-111.53), lower than the 90months (95%CI 72.77-107.23) for LVI negative patients, and the 5-year OS of LVI positive patients was 53.0%, lower than that of LVI negative patients (79.6%). The difference was statistically significant (P=0.005). The 5-year CSS of LVI positive patients was 57.0%, lower than that of LVI negative patients (85.7%, P=0.009). The results of univariate analysis showed that there were statistically significant differences between the two groups (P < 0.05) in exfoliation cytology (P=0.044), hydronephrosis (P=0.015), preoperative fibrinogen level (P=0.003), lymph node status (P=0.014), pathological stage (P=0.001) and grade (P=0.047). Multivariate Logistic regression analysis showed that hydronephrosis (P=0.022), pathological stage (P < 0.001), lymph node status (P=0.025) and fibrinogen level (P=0.019) were independent factors influencing the occurrence of lymphovascular invasion, and the combination of four indexes above was better than any single index. the ROC curve showed that the area under the curve (AUC) of postoperative LVI was the largest when combined with the four predictors, and the AUC was 0.833 (95%CI 0.759-0.907). When the Youden index was 0.594, the sensitivity was 81.1%, and the specificity was 78.3%.

**Conclusion:**

Lymphovascular invasion is related to hydronephrosis, pathological stage, lymph node condition and fibrinogen level. Patients with preoperative hydronephrosis, high pathological stage, lymph node metastasis and high fibrinogen level were at higher risk of lymphovascular invasion.

## Introduction

Urothelial carcinoma can be categorized into upper urinary tract and lower urinary tract divisions. Urothelial carcinoma originating from the pyelocaliceal cavities and ureter is referred to as upper tract urothelial carcinoma (UTUC). UTUC is a relatively uncommon form, accounting for only 5%-10% of all urothelial carcinomas. However, the prognosis for patients with UTUC is poorer compared to those with bladder urothelial carcinoma, with approximately 60% of UTUC patients being diagnosed at an advanced stage (1). Radical nephroureterectomy (RNU) along with bladder cuff resection has long been established as the gold standard treatment for high-risk UTUC, regardless of tumor location (1).

However, the high recurrence rate and poor prognosis following RNU remain significant challenges in the treatment of UTUC (2). Therefore, it is imperative to investigate the prognostic factors and risk of recurrence in UTUC patients. Retrospective studies on UTUC have reported a detection rate of approximately 20% for lymphovascular invasion (LVI) (3), which plays a crucial role in systemic dissemination (4) and negatively impacts patient prognosis. Research has demonstrated that LVI or tumor cell invasion into blood vessels or the lymphatic system may serve as important factors contributing to the unfavorable outcomes observed in solid tumors, such as gastric cancer (5). The growth of UTUC relies on neovascularization for nutritional support, with abundant neovascularization providing a pathway for tumor cells to enter circulation through blood or lymphatic vessels and subsequently metastasize to distant sites. Manoharan M et al. conducted a study involving 357 patients and discovered that LVI was significantly associated with T stage, lymph node metastasis, and tumor grade. Patients with LVI exhibited a markedly higher recurrence rate compared to those without LVI (P < 0.001), as well as a significantly lower long-term survival rate (P < 0.001) (6). Studies conducted by Margulis et al. (7) and Mellouli et al. (8) have also identified LVI as a predictor of postoperative recurrence and poor prognosis in UTUC patients. However, there is currently no clear consensus regarding which factors are more likely associated with LVI. Identifying these factors would be clinically significant as it could help identify patients at higher risk of disease recurrence, enabling early follow-up or adjuvant therapy interventions.

## Patients and methods

143 patients with UTUC who underwent radical surgery at a high-volume center in China between January 2010 and December 2022 were included in this study. Exclusion criteria comprised patients with other primary or secondary malignancies, those with incomplete clinicopathological data or missing follow-up information, and individuals diagnosed with distant metastases prior to surgery. Their age ranged from 49 to 83 years. There were 78 males (54.5%) and 65 females (45.5%). The tumors were located in the renal pelvis in 43 cases (30.1%), in the ureter in 80 cases (55.9%), and in both the renal pelvis and ureter in 20 cases (14.0%). We retrospectively collected the clinicopathologic data from the medical records, including age, sex, body mass index (BMI), occurrence of hypertension, diabetes and hydronephrosis, lymph node invasion, surgical margin, tumor location and side, T stage, tumor grade, as well as laboratory data including exfoliative cytology, fibrinogen levels, absolute neutrophil count, absolute lymphocyte count, absolute platelet count, and albumin levels. Elderly patients were defined according to the World Health Organization's definition of 65 years old or older. The tumor stage was determined based on the American Joint Committee on Cancer (AJCC) staging system. The tumor grade was defined according to the 2004 World Health Organization (WHO) classification system. LVI was defined as wall invasion, destruction, or intraluminal tumor thrombus of the small vein, arteriole, or lymphatic vessel of the tumor (9).

Firstly, all patients were divided into lymphovascular positive and negative according to the postoperative lymphovascular invasion. The median follow-up duration for all patients was 67 months. The study end was overall survival (OS) and cancer-specific survival (CSS) after RNU. The survival of two groups were compared and the clinicopathological characteristics was evaluated. Additionally, the univariate and multivariate binary logistic regression analyses were performed to evaluate the risk factors of lymphvascular positive patients. Lastly, a novel prediction model was established and its diagnostic value was assessed by constructing a receiver operating characteristic (ROC) curve. The study was approved by the institutional review board from The Luoyang Central Hospital Affiliated to Zhengzhou University. Written informed consent was obtained from the involving patient for the publication of this study. In order to confirm the original diagnosis, we invited an experienced urological pathologist to check all the pathological specimens again. Hydronephrosis was defined as abnormal dilation of the renal pelvis and calyces due to obstruction of the urinary system. Preoperative evaluation comprised color Doppler ultrasonography, CT, and/or MRI to confirm the presence of hydronephrosis and to evaluate potential systemic metastasis.

### Statistical analysis

The Kaplan-Meier method was used to estimate the overall survival (OS) and cancer specific survival (CSS) of the patients, and the survival curve was drawn. The log-rank test was used to compare the OS and CSS. The measurement data that did not conform to the normal distribution were expressed as the median (lower quartile, upper quartile), and non-parametric test was used for comparison between groups. The measurement data conforming to normal distribution were described by mean ± standard deviation, and T-test was used for inter-group comparison. The statistical data were expressed as rate (%) and χ2 test was used for comparison between groups. Univariate analysis was employed to examine the risk factors associated with LVI in UTUC patients, while a binary logistic regression model was utilized for multivariate correlation analysis. The results of the multiple factor analysis were used to construct a novel prediction model, and its predictive value was evaluated using receiver-operating characteristics (ROC) curve. Statistical significance was defined as P < 0.05. A two-sided p-value of <0.05 was considered significant. All the statistical analyses were conducted using SPSS version 26.0 (IBM Corporation, Armonk, NY, USA).

## Results

**Figure 1:**
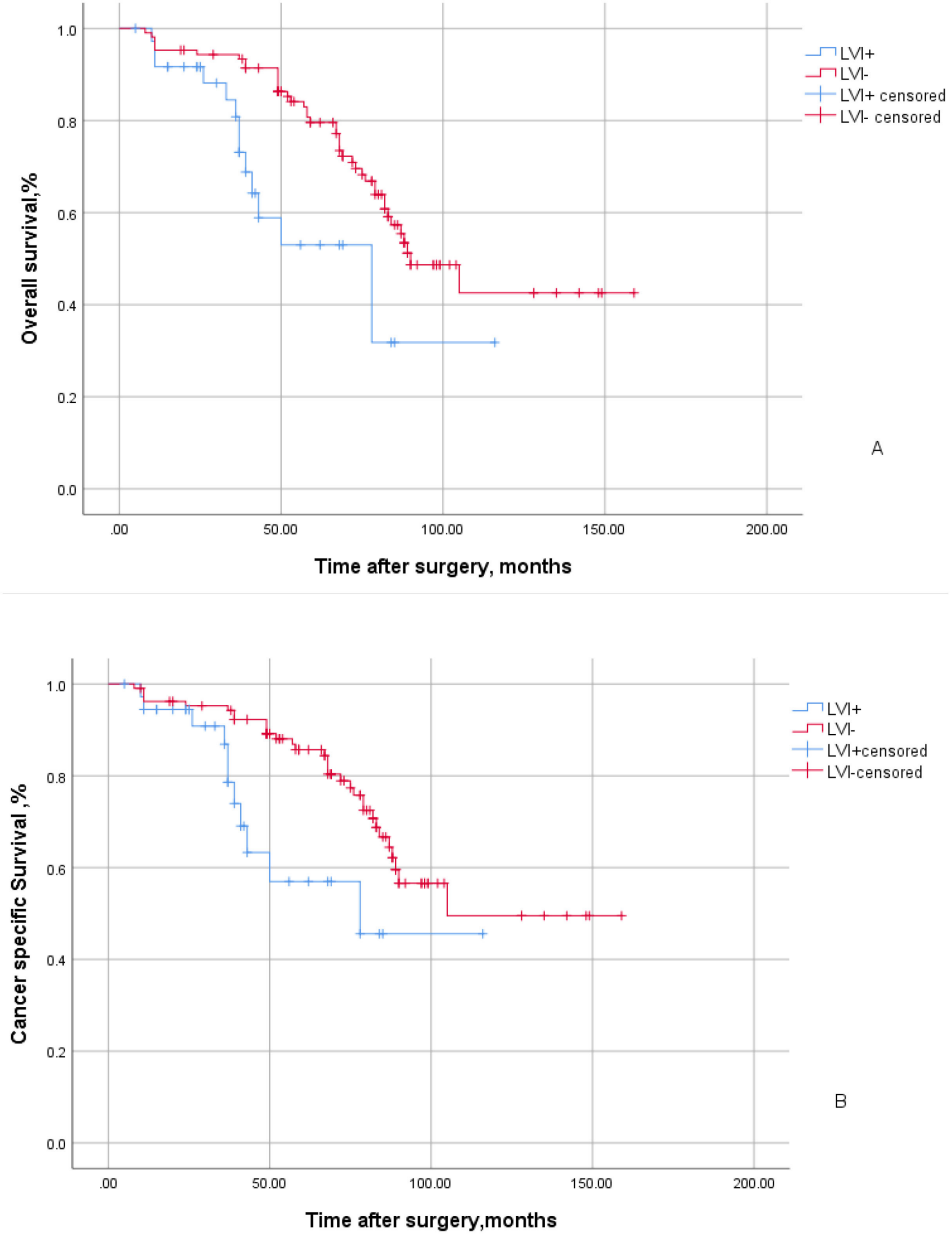
Kaplan-Meier curves of overall survival **(A)** and cancer specific survival **(B)** stratified by LVI in 143 patients with UTUC. The blue line indicates LVI positive status and the red line indicates LVI negative status.

A total of 143 patients with UTUC were included in this study, with 37 cases (25.9%) exhibiting LVI positive and 106 cases (74.1%) showing LVI negative status. A total of 68 patients exhibited positive lymph node status post-surgery. Among them, 18 patients underwent open or intraoperative procedures, while 125 patients received laparoscopic surgery. The median follow-up duration for all patients was 67 months, during which a total of 55 patients succumbed to mortality, including 14 LVI positive patients and 41 LVI negative patients. Among them, tumor specific deaths accounted for the demise of 42 patients, comprising 11 individuals with LVI positive and 31 individuals with LVI negative. UTUC patients with LVI positive exhibited a shorter median survival time at 78 months (95% CI:44.47-111.53) compared to those without LVI involvement (90 months;95%CI:72.77-107.23). The five-year overall survival rate for individuals with LVI positive was lower at 53%, in contrast to the higher rate observed among those without LVI involvement(79.6%). This difference demonstrated statistical significance (P=0.005; **Figure 1A**). Similarly, the five-year cancer-specific survival rate stood at 57% for individuals with LVI positive while it reached 85.7% for those with LVI negative. The statistical significance remained evident(P=0.009; **Figure 1B**).

Among the 143 patients, 78 (54.5%) had comorbid hypertension, 47 (32.9%) had comorbid diabetes, and 72 (50.3%) had comorbid hydronephrosis. To further elucidate LVI positive risk factors, we conducted single factor analysis which revealed no statistical significance (P > 0.05) in age, sex, hypertension, diabetes, BMI, albumin levels, surgical margin, tumor location and side between the two groups. However, a presence of significant differences were observed in positive exfoliative cytology, preoperative hydronephrosis, fibrinogen levels, positive lymph nodes, and pathological stage and grade (P < 0.05) (**Table 1**).

**Table 1:** The clinicopathological characteristics of UTUC patients with LVI positive and LVI negative groups.

Variable	LVI positive (n=37)	LVI negative (n=106)	*x* ^2^/*t*/*z*	*P*
Age (yr)			0.873	0.350
≥65	22 (59.5)	72 (67.9)		
<65	15 (40.5)	34 (32.1)		
Sex			1.489	0.222
Male	17 (45.9)	61 (57.5)		
Female	20 (54.1)	45 (42.5)		
Hypertension			0.700	0.403
Yes	18 (48.6)	60 (56.6)		
No	19 (51.4)	46 (43.4)		
Diabetes			1.651	0.199
Yes	9 (24.3)	38 (35.8)		
No	28 (75.7)	68 (64.2)		
BMI/kg·m^-2^	23.9 (22.6, 25.4)	23.8 (22.7, 25.0 )	-0.104	0.917
Exfoliated cells			4.057	0.044
Positive	21 (56.8)	40 (37.3)		
Negative	16 (43.2)	66 (62.3)		
Hydronephrosis			5.919	0.015
Yes	25 (67.6)	47 (44.3)		
No	12 (32.4)	59 (55.7)		
Tumor side			0.349	0.555
Left	21 (56.8)	66 (62.3)		
Right	16 (43.2)	40 (37.7)		
Albumin /g·L^-1^	41.75 ± 4.67	41.62 ± 4.63	0.143	0.886
Fibrinogen /g·L^-1^	3.670 (2.870, 3.975)	2.815 (2.490, 3.775)	-3.009	0.003
NLR	2.830 (2.211, 3.337)	2.531 (2.078, 3.005)	-1.692	0.091
PLR	133.188 (96.538, 157.941)	113.100 (92.370, 127.049)	-1.927	0.054
Tumor location			0.211	0.900
Renal pelvis	11 (29.7)	32 (30.2)		
Ureter	20 (54.1)	60 (56.6)		
Both	6 (16.2)	14 (13.2)		
Surgical margin			0.226	0.635
Positive	12 (32.4)	30 (28.3)		
Negative	25 (67.6)	76 (71.7)		
Lymph node involvement			5.999	0.014
Positive	24 (64.9)	44 (41.5)		
Negative	13 (35.1)	62 (58.5)		
pT stage			10.219	0.001
≤T2	10 (27.0)	61 (57.5)		
>T2	27 (73.0)	45 (42.5)		
Tumor grade			3.949	0.047
High	22 (59.5)	43 (40.6)		
Low	15 (40.5)	63 (59.4)		

Neutrophil-to-lymphocyte ratio (NLR), Platelet-to-lymphocyte ratio (PLR).

In order to further investigate the risk factors for lymphovascular invasion (LVI), we conducted a multivariable logistic regression analysis, including the following indicators: exfoliated cytology, presence of hydronephrosis and lymph node metastasis, fibrinogen level, tumor grade, and pathological stage. The results revealed significant associations between preoperative hydronephrosis, advanced pathological stage, lymph node metastasis, and fibrinogen level with LVI positivity. However, no significant correlations were observed between pathological grade or exfoliative cytology and LVI positivity (**Table 2**).

**Table 2:** Multivariate analysis of LVI positive UTUC patients.

Covariates	B	*P*	OR	95%*CI*
Hydronephrosis	1.139	0.022	3.123	1.176-8.289
pT stage	1.756	<0.001	5.788	2.193-15.275
Lymph node metastasis	1.055	0.025	2.872	1.140-7.232
Fibrinogen	0.859	0.019	2.361	1.152-4.841
Tumor grade	0.839	0.067	2.314	0.943-5.677
Exfoliated cells	0.818	0.071	2.266	0.932-5.511

**Figure 2:**
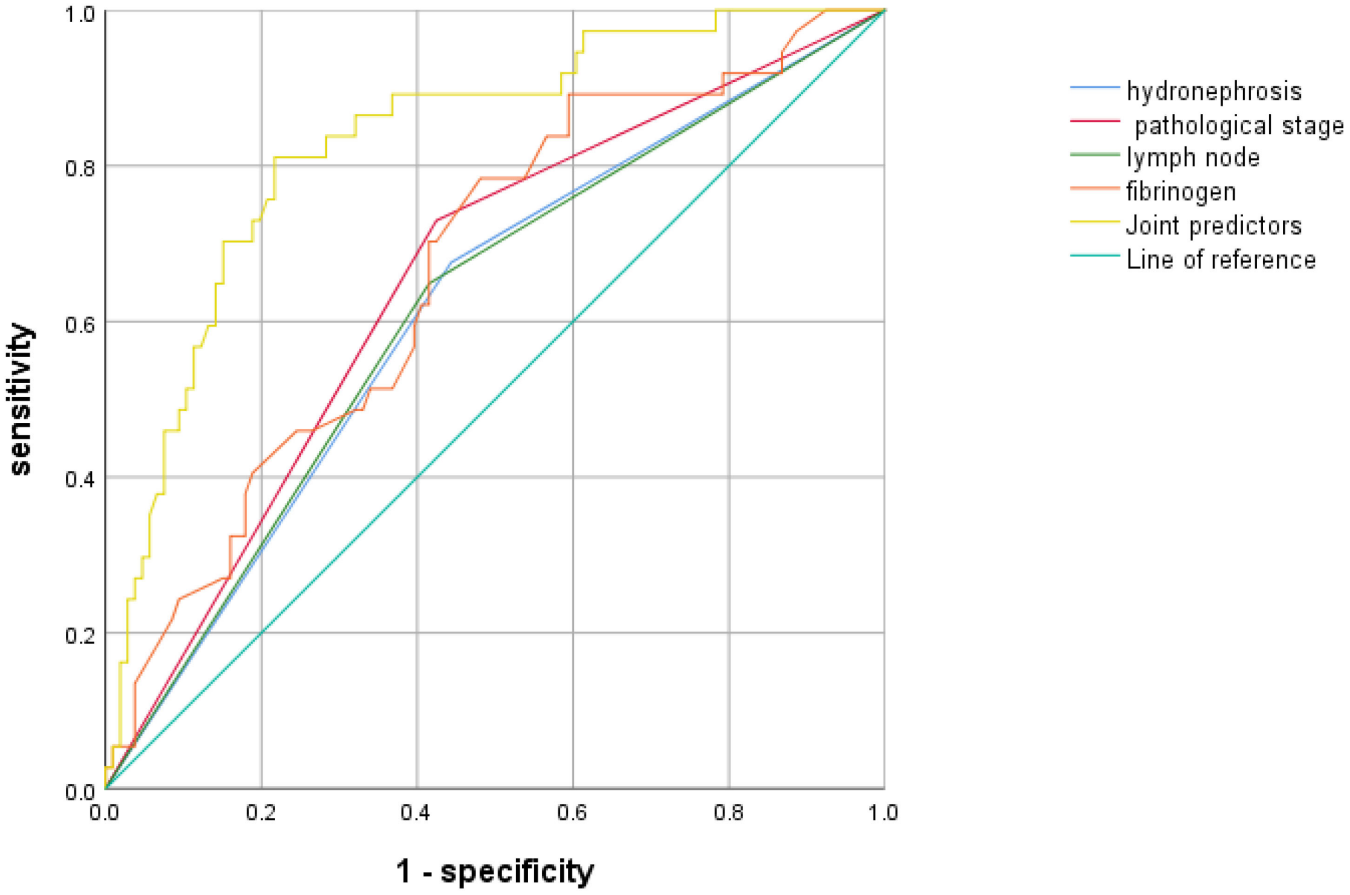
The predictive value of each predictor and joint predictor for LVI positivity.

A joint predictor was established using four independent risk factors, including hydronephrosis, lymph node metastasis, pathological stage, and fibrinogen level, to predict positive LVI after UTUC. A ROC curve was constructed and the largest area under the curve (AUC) for the joint predictors was found to be 0.833 (95%CI: 0.759-0.907, P < 0.001). The maximum Youden index was 0.594, with a sensitivity of 81.1% and a specificity of 78.3%, indicating that the combination of the four predictors had a high predictive value for positive LVI after UTUC. (**Figure 2**)

## Discussion

In recent years, UTUC has emerged as a prominent research focus in the field of urology both domestically and internationally. The absence of symptoms and delayed diagnosis often result in muscle invasive or locally advanced tumors (56%) at the time of presentation, leading to a lower survival rate compared to bladder urothelial carcinoma. Patients with invasive UTUC involving the muscle layer, specifically T2 and T3 stages, have a 5 year survival rate below 50%, whereas those with T4 stage exhibit less than 10% survival rate (10). Therefore, it is crucial to identify prognostic factors associated with UTUC for risk stratification and effective disease management.

In our study, the rate of lymphovascular invasion (LVI) positivity was 25.9%, which is consistent with the LVI detection rate of 25% reported in previous studies (11, 12). Furthermore, numerous prognostic factors associated with disease recurrence or survival have been identified in prior research, among which LVI plays a pivotal and significant role in tumor dissemination (13). The prognostic value of LVI has been documented in various urological malignancies including penile cancer, prostate cancer, and bladder cancer (14). Recently, there have been relevant studies investigating the correlation between LVI and prognosis in patients with UTUC (15). Cancer growth is frequently associated with an expansion of lymphatic vessels (lymphangiogenesis) within and surrounding the primary tumor (histologically most frequently detected with the D2-40 anti-podoplanin antibody) and with lymphangiogenesis in tumor-draining lymph nodes. A large number of experimental and clinical studies have clearly demonstrated that the density of tumor-associated lymphatic vessels, the detection of lymphatic invasion by tumor cells, and intratumoral expression of the main lymphangiogenic growth factor VEGF-C all correlate with lymph node metastasis and poor patient outcome in many cancer types (16). Elenkov AA et al. (17) and Kamai T et al. (18) have highlighted that LVI serves as an independent prognostic factor for overall survival in UTUC patients following surgery. These findings align with the results of our study, emphasizing the importance of elucidating the risk factors associated with LVI in postoperative patients' prognosis.

Previous studies have demonstrated that LVI is associated with prognostic factors identified in UTUC, such as advanced tumor stage, higher pathological grade, and tumor necrosis (19, 20). A meta-analysis revealed a significant correlation between LVI and poor cancer-specific survival, overall survival, and recurrence-free survival. Furthermore, LVI was significantly associated with advanced tumor stage, higher pathological grade, and lymph node metastasis (21). Similarly, Lee HY et al. (22) reported a significant correlation between LVI and tumor stage and grade. In this study of 143 UTUC patients, there were 37 cases of LVI positive patients with pathological stage > T2; among them, 27 cases (73.0%) had high-grade tumors (59.5%). Among the 106 cases of LVI negative patients with pathological staging > T2, there were a total of 45 cases (42.5%) with high grade tumors (40.6%). Univariate analysis indicated a significant difference between LVI positive and LVI negative patients (P=0.001). Multivariate analysis further confirmed that pathological stage remained significantly correlated with LVI positivity (P < 0.001).

The presence of lymph node metastasis has been identified as a significant indicator of tumor progression in previous studies. Li X et al. found that a total of 73 patients (18.7%) had local recurrence within a median follow-up of 41 months (range 3-80 months), and multivariate analysis showed that lymph node metastasis was an independent predictor of increased local recurrence (23). Sato R et al. (24) discovered that the presence of lymph node metastasis was an independent prognostic factor for poor overall survival in 68 patients with UTUC who underwent radical nephroureterectomy (RNU). Milojevic B et al. (25) demonstrated that lymph node metastasis independently predicted reduced tumor specific survival. The results of both univariate and multivariate analyses revealed a significant association between LVI and lymph node status (P < 0.05), suggesting that lymph node metastasis can serve as a risk factor for postoperative LVI in UTUC patients.

In patients with bladder cancer, Stimson CJ et al. (26) reported that concomitant hydronephrosis before radical cystectomy was an independent predictor of adverse pathological features. Messer JC et al. (27) and Chung PH et al. (28) also reported this relationship in their separate studies of patients with high-grade UTUC. Sakano S et al. (29) found a significant correlation between positive urine cytology and hydronephrosis and LVI through their study. Ito Y et al. (30) found that the higher the degree of hydronephrosis, the higher the tumor pathological stage and the positive LVI were significantly correlated. This was consistent with the conclusions drawn in this paper. The incidence of preoperative hydronephrosis was 25 cases (67.6%) among the 37 patients with lymphovascular invasion positive, while it was 47 cases (44.3%) among the 106 patients with LVI negative patients. Both univariate analysis (P=0.015) and multivariate analysis (P=0.022) demonstrated a statistically significant difference. Qian S et al. (31) propose that hydronephrosis can be attributed to luminal obstruction, intraluminal infiltration, or exogenous compression. Consequently, hydronephrosis can result in outward expansion and longitudinal thinning of the upper urinary tract, thereby facilitating the dissemination of cancer cells to local or distant organs. Another study also demonstrated a higher likelihood of developing high grade tumors and lymphovascular invasion positive following radical nephroureterectomy treatment in patients with positive urine cytology (32). The positive preoperative exfoliative cytology was observed in 21 out of 37 patients (56.8%) with lymphovascular invasion (LVI) and in 40 out of 106 patients (37.3%) without LVI. Univariate analysis (P=0.044) demonstrated that urine exfoliative cytology independently increased the risk of positive LVI. However, multivariate analysis showed no statistically significant difference between urine cytology positivity and LVI occurrence (P = 0.071). Further validation is required in a multi-institutional UTUC database to establish the association between urine cytology and LVI.

In conducting research on the risk factors associated with LVI positivity, in addition to typical clinical and pathological factors such as pathological staging classification, other factors including clotting factor inflammation, malnutrition, and systemic influences have been reported to significantly impact tumor occurrence and development (33). This study incorporates the analysis of fibrinogen levels, albumin levels, NLR (neutrophil-to-lymphocyte ratio), and PLR (platelet-to-lymphocyte ratio). Both univariate and multivariate analyses demonstrate that fibrinogen level is a statistically significant factor. Firstly, fibrinogen can accumulate around cancer cells, promoting their proliferation and serving as a source of certain growth factors such as vascular endothelial growth factor and fibroblast growth factor, which facilitate tumor angiogenesis (34). Secondly, fibrinogen and its degradation products can enhance platelet adhesion to cancer cells, thereby promoting cancer metastasis. Consequently, the ascending levels of fibrinogen can serve as an indicator of tumor inhibition, while a persistent increase in fibrinogen levels often suggests the potential for tumor metastasis (35). Additionally, the hematological marker fibrinogen holds clinical value due to its affordability and easy accessibility.

However, The present study has certain limitations. The first limitation lies in the inherent constraints of a single-center retrospective study. Secondly, even after employing rigorous multivariate analysis to control for potential confounding factors, it is important to acknowledge that complete elimination of selection bias may not have been achieved. The rarity and low incidence of UTUC necessitate further expansion of the number of risk factors and sample size, as well as the need for multi-center prospective studies to provide additional confirmation and obtain more precise conclusions.

## Conclusion

After conducting research, we found that the hydronephrosis, high pathologic stage, lymph node metastasis, and high level of fibrinogen were identified as independent risk factors for LVI positivity. This study provides clinicians with valuable insights to assess the condition of UTUC patients with LVI and identify those at a higher risk. Early preoperative management and auxiliary treatment based on these findings hold significant clinical significance.

## Data Availability

The original contributions presented in the study are included in the article/supplementary material. Further inquiries can be directed to the corresponding authors.
